# The Amendment Act

**Published:** 1913-08-30

**Authors:** 


### NATIONAL INSURANCE ACT DEPARTMENT.

### LXV.—The Amendment Act.

National Insurance against loss of health and for the prevention and cure of sickness in this country is now governed by two Acts of Parliament, for the Amendment Bill became an Act on August 15 last under the short title of the National Insurance Act, 1913, and the two Acts together will be cited as the National Insurance Acts, 1911 to 1913. The word "to" joining the years 1911 and 1913 looks ominously suggestive of further amending Acts, for when such Acts become incorporated with the present measures it will only be necessary to alter the terminal date without specifically referring to the intermediate amending Acts. In its final form the National Insurance Act, 1913, does not differ materially from the Amendment Bill as it passed third reading in the House of Commons. Drafting amendments only were made by the House of Lords, and these were agreed to in due course by the Commons.

The introduction of the measure in the Commons was so long delayed that the Bill did not have the advantage of an exhaustive review by the Upper Chamber, as it had to pass all its stages in that House within the last crowded week of the Parliamentary Session.

### One Section only Operates at Once.

Although the Act became law on August 15, only one of its sections became operative on that date. This section (Section 29) is not of much interest to insured persons or to the public generally, but is of a technical character, as its principal object is to confer on the Joint Committee of the Insurance Commissioners the same powers of issuing valid documents and Orders as were conferred on the separate Insurance Commissioners by the Act of 1911. The second sub-section is in fact retrospective in its action, for it gives validity to documents already issued by the Joint Committee in excess of their strict legal powers under the principal Act.

The section was made, by a drafting amendment inserted by the Lords, to come into operation on the passing of the Act, in order that certain documents which will have to be issued by the Joint Committee before certain of the remaining sections of the Act come into operation may have legal effect and sanction.

Apart from this section, it is provided in Subsection 3 of the last section (Section 43) that except as otherwise expressly provided the Act shall come into operation on September 1, 1913, or such later date or dates as the Joint Committee may by Order appoint, and powers are given to the Committee to appoint different dates for the commencement of the different provisions of the Act. The limiting date beyond which none of the provisions of the Act may remain inoperative is January 15, 1914.

A circular has just been issued (Circular A.S. 103) by the Insurance Commissioners to approved societies setting forth particulars of the most important provisions of the Amendment Act, and giving the dates on which they are to come into operation.

### Abolition of Reduction of Benefits.

The principal date to be borne in mind is October 13, 1913, for that is the date expressly specified so that the provisions of the Act altering the rates of benefit may not be longer deferred.

Monday, October 13, will be the first day of the sixth quarter, and new Insurance Cards and Insurance Books will become current as from that day. It will, therefore, be the most suitable opportunity for varying the present rates of benefit, and is the date which has been selected for the purpose.

The abolition of the reduction of benefits to insured persons who are employed contributors and were above fifty years of age on first becoming insured is one of the most important alterations made by the new Act. It is the most costly of the alterations. According to the actuarial estimates it will involve an addition to the initial reserve values of over three million three hundred thousand pounds, and will defer the redemption of those reserves for a period estimated at nearly thirteen months. Furthermore, the additional charge to the State in the year 1915 will be about £160,000, this amount being slightly reduced from year to year subsequently as the old persons affected gradually pass out of insurance.

### Additional Benefits and the Young.

While we do not grudge the insured persons in advancing years the extra benefits provided, we feel that it is important that the means whereby the grant has been made should be fully appreciated. In large measure the extra benefits will be provided out of the reserves, and it is the younger insured persons principally who contribute to make those reserves an accomplished fact. Thus it is clear that in the main the Government has been generous to the old persons at the expense of the young ones; and it will, in addition, be necessary to call upon the taxpayers for a substantial sum each year for many years to come. Until the initial reserves have been redeemed it is impossible for young persons now entering insurance at the age of sixteen to obtain the ninepennyworth of benefit that it is generally supposed was promised by the Chancellor of the Exchequer for their weekly contribution of 4d. As matters now stand it is estimated that over twenty years will elapse before the reserve is made up by the sinking fund, and not until then can the deduction for sinking-fund purposes from the contributions be dispensed with.

The young people are, of course, obtaining the benefit of the employers' contributions as well as their own—that is to say, in no case is the value of the benefit less than 7d. for each 4d. paid in contribution by the worker; and it may, therefore, be argued that the young people have no cause to complain. The most disconcerting feature is, however, that the means has been discovered whereby additional benefits can be given to one class of insured persons at the expense of another class, and further, that in connection with the operation of the new scheme regarding arrears of contribution, the sinking fund may itself be depleted of a substantial sum each year. Further developments may be desired in the future, and it will be a great temptation to coming Chancellors of the Exchequer to provide the means by a similar process. Twenty years is surely long enough for the full benefits of the Act to accrue to the young persons, and any further extension of the period will have to be guarded against at all costs. The operation of the sinking fund and the necessity for the initial reserves are in fact very simple to explain, but they are not generally understood, possibly through the use of technical terms. For this reason it is all the more important that those who really understand that the plan of finance involves the application of the State subsidy to providing benefits to the old persons in much greater proportion than to young persons should not fail, when opportunity serves, of making the facts more widely known.

### The Necessary Alterations.

So far as the Insurance Commissioners are concerned the abolition of the reduction of benefits will necessitate the re-calculation of the reserve values of insured persons over fifty years of age at entry into insurance as employed contributors. It will also be necessary for alterations to be made in the sums credited to the individual approved societies in the books of the respective Commissions. The approved societies will at first be caused some extra trouble. Rates of benefit entered on their records will require alteration. Subsequently to this initial disturbance in routine the alteration will conduce to smoothness of administration, as it will remove one of the complications in paying the benefits when they become due. Promptitude in settlement of claims is one of the principal requirements of a satisfactory system of insurance of the industrial population, and any amendment of the Act which, by removing difficulties, enables the payments to be made promptly is to be welcomed.

### The Time for Entry Extended.

Monday, October 13, 1913, is an important date for another reason, for by Section 2 the time allowance of one year from the commencement of the principal Act, which was allowed by that Act for entry into insurance at ages above sixteen at full rates of benefit, is extended by a further thirteen weeks. Thus, a further opportunity is afforded to those who have until now been content to remain as deposit contributors of becoming fully insured as members of approved societies. Many deposit contributors have already experienced the disappointment of being informed of the inadequacy of the fund standing to their credit to pay the benefits when ill which they would have obtained as members of approved societies. The matter is now more fully appreciated, and it is to be hoped that deposit contributors as a class will take advantage of the concession.

There are others who may, and should, in many cases take advantage of the extension of time; we refer, of course, to those who have obtained certificates of exemption from insurance. An exempted person is one who comes within the compulsory provisions of the Act, and whose employer has to pay his own contribution, but who obtains a certificate exempting him from paying the worker's contribution on one or other of certain specified grounds. These are—that he is in receipt of a pension or income of £26 a year or upwards, not dependent upon his personal exertions, or that he is ordinarily or mainly dependent upon some other person, or, as added by the new Act, is ordinarily and mainly dependent on the earnings derived from other occupation. Many such persons could, with great advantage to themselves, pay their own contribution and thus obtain the full benefits of the insurance. They now have until October 13 in which to decide.

The extension of time for entry at full rates of benefit will also operate to the advantage of those who become employed within the meaning of the Act for the first time within the thirteen weeks ending on October 12 next.

### A Concession to Voluntary Contributors.

Voluntary contributors are also given the same concession. In their case the period for entry at the flat rate of contribution up to the age of forty-five, and the special rate at higher ages only extended under the principal Act for six months after it came into operation. The time, in their case, has already expired some time since, and it is presumed that if any voluntary contributors have joined at the higher rates that have been charged since January 15 last they will be given the opportunity of re-entry at the lower rate which is now re-opened to them until October 12. The Act makes no mention of retrospection in this connection; and, presumably, there will be no possibility of claiming excess payments. The class of voluntary contributor is very much smaller than that originally estimated, and it is possible that few, if any, have entered since the full rates of contribution became operative.

---

NOTE.—Questions and Correspondence on all doubtful points are invited, and should be addressed to the Editor, The Hospital, 28, 29 Southampton Street, Strand, London, W.C., and marked "Insurance."

